# Development and Validation of a Railway Safety System for Nordic Trains in Isolated Territories of Northern Quebec Based on IEEE 802.15.4 Protocol

**DOI:** 10.3390/s21186129

**Published:** 2021-09-13

**Authors:** Laurent Ferrier, Hussein Ibrahim, Mohamad Issa, Adrian Ilinca

**Affiliations:** 1 Wind Energy Research Laboratory, Université du Québec à Rimouski, Rimouski, QC G5L 3A1, Canada; Adrian_Ilinca@uqar.ca; 2 Institut Technologique de la Maintenance Industrielle, Sept-Îles, QC G4R 5B7, Canada; Hussein.Ibrahim@itmi.ca; 3 Institut Maritime du Québec à Rimouski, Rimouski, QC G5L 3A1, Canada; missa@imq.qc.ca

**Keywords:** isolated environment, IT model, zigbee, IOT, doppler effect, fading channel, AWG, FSK, GPS, nordic trains, connected watch

## Abstract

Connected objects are deployed all over the world. Thus, they are contributing to improving communications. In urban areas, technological challenges are gradually being overcome, and advances in this area are exponential. Unfortunately, isolated territories such as northern Quebec do not beneficiate from this technological progress. Yet, northern Quebec relies on abundant natural resources, with notably its huge hydroelectric dams and iron mines, and therefore, the region’s economic life revolves essentially around the exploitation of these resources and is heavily reliant on rail transportation. However, according to Transport Canada, 1246 railroad accidents were reported in 2019 to the Transportation Safety Board (TSB). Thirty-eight people described as trespassers lost their lives, and five railroad employees were fatally injured. In this context, we present the implementation of a security system in an isolated environment for employees intervening on the railroad track to warn them of the imminent arrival of a train. Due to the context of the isolated environment, i.e., without an electrical network, without internet, and without an LTE network, a solution for employees has been developed using a Zigbee telecommunication system and a connected watch. A case study on a train operating in a remote and isolated area in northern Quebec is presented to validate the performance of the proposed system based on an open-source and customizable solution.

## 1. Introduction

Northern Quebec relies on its abundant natural resources, notably its energy, mineral and iron resources, and forestry. The region’s economic life revolves around exploiting these resources and is heavily reliant on rail transportation.

**Figure 1:**
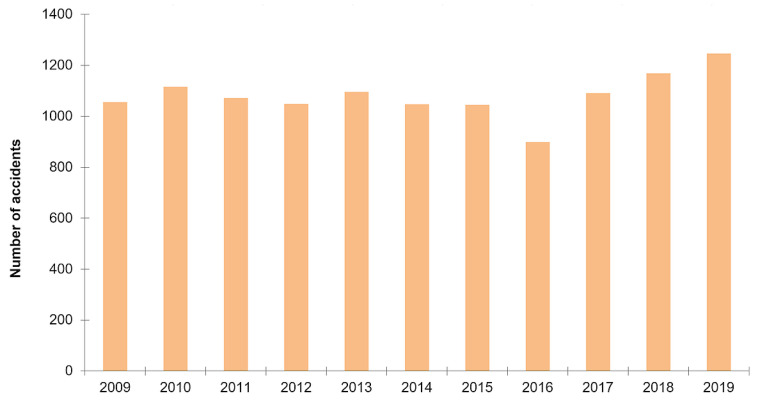
Number of rail accidents in Canada, 2009 to 2019 [1].

Of all the railway networks in the world, Canada has the third largest and transports the fourth most significant volume of goods. Every year, Canadian railways move 70% of the country’s surface goods (including 40% of its exports) and carry 70 million people, meeting motorists and pedestrians at thousands of public and private crossings [1]. In 2019, 1246 rail accidents were reported to the Transportation Safety Board (TSB), which was up from 1169 in 2018. It represents a 17% increase from the previous 10-year (2009–2018) average of 1064 (Figure 1) [1].

**Figure 2:**
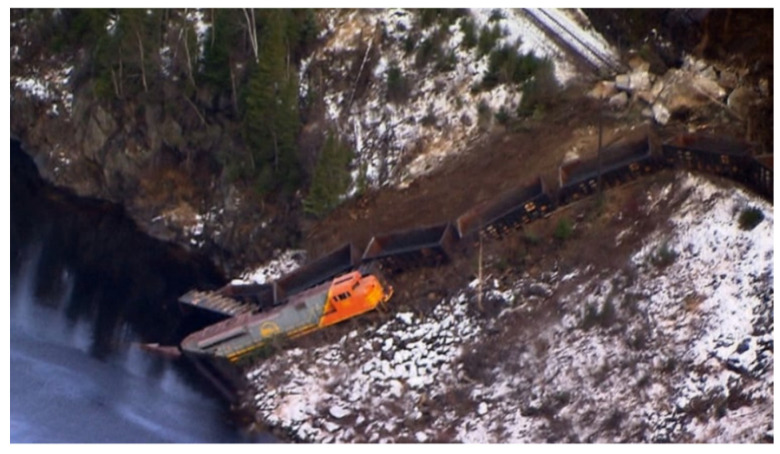
The derailment of the train in the Moisie river.

The number of deaths due to railway accidents has also increased by 25% between 2018 and 2019 [2]. One of the most famous rail disasters in Canada was the Lac-Megantic accident in 2013 in which 47 people died [3]. In northern Quebec, railway convoys can contain 200 wagons full of iron ore: therefore, the convoys’ length is very important, and it takes about 2 km to stop the train. The most common railway accidents in those sectors are due to rockfall on the tracks or the railway tracks structure defaults because of the very hard winter conditions. The proof: the derailment of the QNS & L train on 6 November 2014, following a landslide that the driver saw too late. The train descended a slope to dive into the Moisie River (northeast of Sept-Îles, QC, Canada), causing the driver’s death (Figure 2).

Within this context [4], Transport Canada defined objectives to improve rail safety, increase safety at grade crossings and along rail lines, and increase public confidence in Canada’s rail transportation system. As a solution to this problem, our article’s objective is to present with a scientifical process the development and the validation of a railway safety system for isolated environment and based on radio waves using ZigBee telecommunication protocol. Our contribution and the beauty of our paper is to associate known concepts that have never been associated such as ZigBee technology and Nordic freight trains in an isolated environment where internet of things, LTE, and more generally, technologies usually used in cities do not have a place right now. The system was implemented on a train linking two cities on the north coast of Quebec in an isolated environment to assess its performance and demonstrate its viability.

The paper is structured as follows: Section 2 presents the isolated environment as the important context of the developed solution. Section 3 presents the technological choices for the radio protocol, while Section 4 presents the mathematical models and simulation with MATLAB and Radio Mobile software. Section 5 presents the case study: it describes the design decisions, the system integration embedded on board as well as the connected watch design and finally, the tests process and the results obtained. Finally, Section 6 concludes the paper with future prospects of study in this area.

## 2. The Context of the Solution: The Isolated Environment

**Figure 3:**
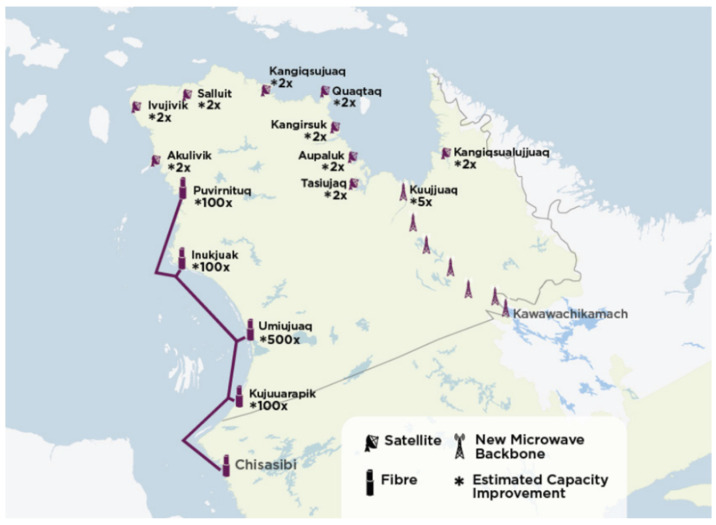
Telecommunication network in the Nanavut.

About two hundred thousand people are living in isolated regions in northern Canada. The Nunavut, located in the north of Quebec, is a good example of what is an isolated place. The Quebec government has invested a lot of money with a grant called “le plan nord”, but nevertheless, the telecommunication network stays almost non-existent (Figure 3): the optical fiber and telecommunication towers have been deployed in some places, but it is very far from the totality of the territory. The only solution in some places is the satellite but at a very high cost. The railway security system that has been developed is dedicated to trains transporting iron ore from mines located in northern Quebec to the port of Sept-Iles where it is shipped by boats to other countries for processing. Most of the railways in this region do not have any electrical network or any optical fiber network. In the same way, there is no LTE network. Those considerations lead to eliminating technical solutions such as LoRa or Sigfox used in the internet of things systems, which need an internet connection, but also LTE solutions. Note that the trains are freight trains often bought in the USA and adapted for very hard climate conditions for the north of Quebec. Those trains are very poor technologically, and so, the electronic solution of the system developed must be adapted to those trains. Therefore, it has been a very high challenge level to develop the solution presented further in this paper, which is based on ZigBee protocol.

## 3. Selection of Suitable Radio Frequency Protocol

The northern regions of Quebec have an arctic climate with very cold winters and short, much cooler summers. As a result, the electronic components must be carefully chosen. Furthermore, winter train operations in northern Quebec are marked by slower speeds, exposing electronic systems and freight to more extended periods in temperatures as low as −40 °C.

**Figure 4:**
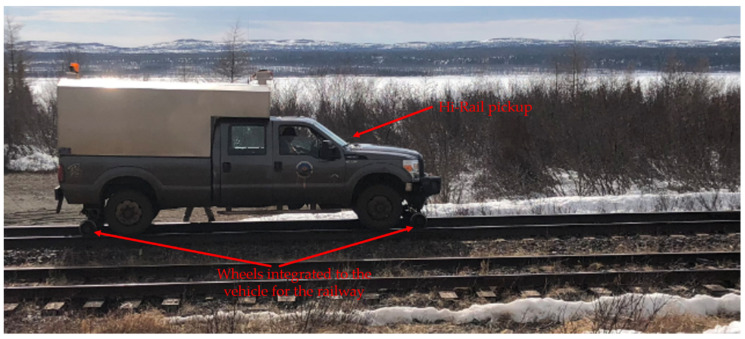
Hi-Rail pickup from Tshiuetin company in northern Quebec.

Most of the railway maintenance employees are called upon to work on the railway track to ensure site maintenance: the necessary equipment is loaded on board a truck moving on the railway track (Hi-Rail pickup) (Figure 4), which drives the employees to the site of the intervention.

While the employees are busy carrying out maintenance operations around the Hi-Rail pickup, one is in radio contact with the nearest train to alert the employees to get to safety when a convoy is coming. Therefore, the idea was to add a second layer of security for the employees with an automatic alert system. Note that the Hi-Rail pickups do not have any geolocate systems: these are rudimentary trucks. The solution consists of installing a transmitter in the locomotive pulling the wagons and a receiver in the Hi-Rail pickup. The locomotive’s transmitter gets the locomotive’s GPS coordinates using a GPS board and sends them wirelessly to the Hi-Rail pickup, which also acquires its own GPS coordinates. Then, it can calculate the distance between the train and itself. Finally, the receiver sends wirelessly this distance to a watch equipping the employees, which converts the distance information into a visual and audio alert that evolves when the train approaches. Before describing the solutions, it is necessary to study the GPS coverage in the isolated place where the Nordic trains run.

### 3.1. Global Positioning System (GPS) Coverage

**Figure 5:**
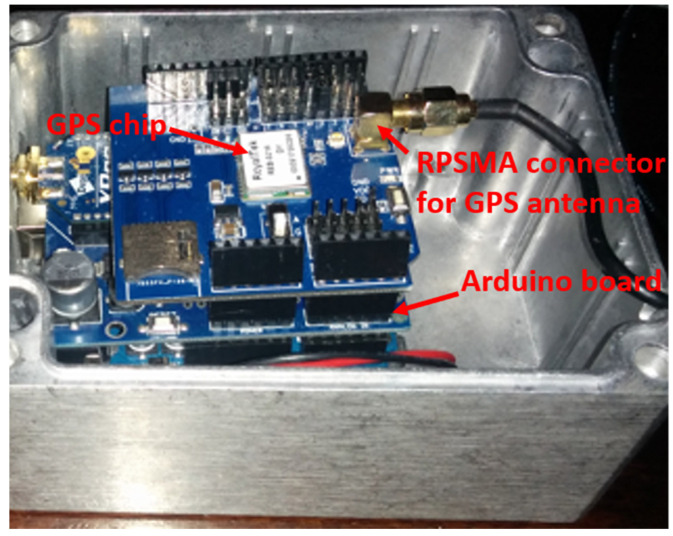
The embedded GPS system on the train.

The viability of the solution described above depends on the GPS coverage on the track of the rail convoy. If the GPS coverage is not sufficient, this solution cannot be adopted. An ATMega 328 microcontroller board associated with a GPS board and a micro-SD card (Figure 5) have been embedded on the train. The system records the GPS coordinates acquired during the journey of the rail convoy every 2 s on the railway where the final solution will be tested.

**Figure 6:**
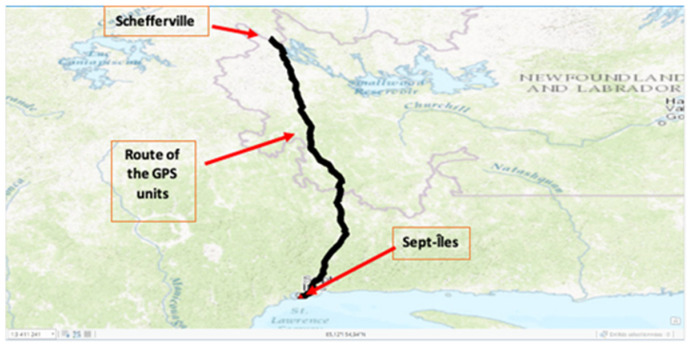
GPS coordinates mapping between Sept-Îles and Schefferville (isolated environment).

**Figure 7:**
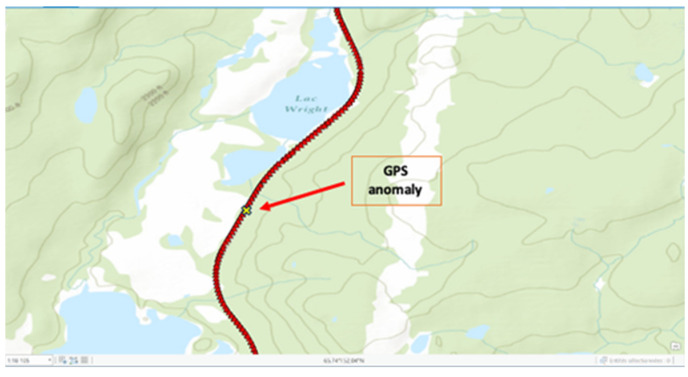
An 86 m distance GPS anomaly toward Mounts Wright.

Over time, a GPS covering map was built over the train journey (Figure 6). A quick calculation showed 99.8% GPS coverage of the train between the two destinations. The areas where GPS coordinates could not be acquired (Figure 7) have been identified as tunnel passages. These results are excellent and have confirmed the relevance of this solution for this railway safety system.

### 3.2. Choice of the Wireless Radio Protocol Solution

The state of the art of railway security systems for Nordic Quebec trains in isolated environments brings only one technology: the PDD (Proximity Detection Device) by QNS&L railway company (Sept-Îles, QC, Canada). It was implemented in July 1997. It uses GPS technology to provide audible and visual alerts. It is an old system, and QNS&L does not provide technical details about its system. Its price is very high, and it is not possible to implement others new functionalities depending on the needs of a railway company.

Therefore, it is necessary to develop our own solution: they are several wireless technologies that could be used to design our railway security system solution:

- LoRa (with LoRaWAN network) or Sigfox;
- LTE;
- Wifi;
- ZigBee;
- Z-Wave;
- Bluetooth;
- Satellite.

The characteristics of these technologies are summarized in the Table 1 below.

The solutions used to communicate in those isolated environment are principally with basic radios or with satellite for big companies. The satellite solution cannot be the technical solution chosen because of the high prices of satellite subscriptions in Quebec. Each of the solutions in Table 1 has their own properties in terms of range, transmission rate, and power consumption, for example. The first one, LoRa [5], is dedicated to the internet of things technology. The North American frequency is 915 MHz, which gives a nice range: the LoRa protocol radio gives about a 25 km range due to a very low transmitted power, with only 25 mW and a spreading factor equal to 12. With this spreading factor, this means that the range is privileged, and therefore, the transmission rate is very low: about 366 bps and the battery life could be around 5 years by node. Results are nearly the same for Sigfox technology [6]. The main problem that eliminates this solution is the isolated environment: on the railway track, there is no optical fiber bringing a connection to internet to push data from the gateway to the cloud. In the north of Quebec and especially near the railway tracks, base stations are non-existent: therefore, the LTE solution is not suitable. Concerning Wifi solutions (Wifi 1 and 2), the data rate is high, but the range is very low. The conclusion is the same for Bluetooth solutions with a range much lower than with Wifi. Therefore, the only solution suitable for isolated environments with a high range is the ZigBee IEEE 802.15.4 radio protocol with XBee radio-frequency boards in the UHF domain and especially a carrier frequency of 915 MHz.

Simulations were performed with Radio Mobile software to specify the ZigBee range at 915 MHz in the region where the system is supposed to be test. Note that this software uses the Longley Rice model [7], which is a geographical model that takes into account the environmental parameters.

**Figure 8:**
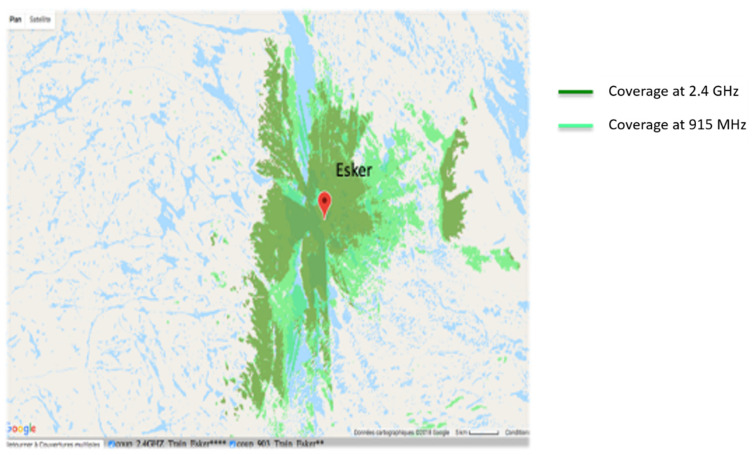
Illustration of the radio coverage simulation at Esker camp (latitude: 53.874178; longitude: −66.419625) on the railway between Sept-Iles and Schefferville.

Coverage studies were carried out at various points along the rail track between Emeril and Schefferville in northern Quebec. The simulations were performed with a carrier frequency set at 915 MHz for the North American UHF domain and also to have a comparison at 2.4 GHz. The receiver sensitivity was fixed at −100 dBm. Later, during the system tests, the omnidirectional antenna on the train will be fixed on the locomotive roof at the height of 15 feet (approximately 4 m), while the receiver antenna will be fixed on the Hi-Rail pickup roof at the height of 2.5 m: therefore, these heights have been used for the simulations. The results of the simulations on the Radio Mobile software (one of those simulations, Figure 8) show that the choice of the carrier frequency at 915 MHz is judicious because the area covered is large enough, and without any surprise, it is larger than with a carrier frequency of 2.4 GHz. The technological choice for radio frequency modules at the transmitter and receiver is based on XTends [8] modules using the Zigbee IEEE.802.15.4 [9] protocol with a carrier frequency of 915 MHz.

The modulation used with XTend boards is of the non-coherent Frequency Shift Keying (FSK) type. However, before continuing with the solution development, we need to qualify the system performances more accurately with the XTends radio protocol parameters. Therefore, detailed telecommunication system modeling is necessary to simulate the effects of the environment where this solution will be implemented.

## 4. System Model

### 4.1. Loss Path (LP) Model

**Figure 9:**
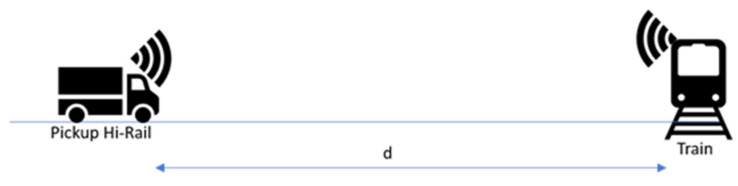
Train–Hi-Rail pickup (LOS).

There are many models to simulate a telecommunication channel. The first one is the LP [10] model, which defines the range of a system when the transmitter and receiver are in line of sight (LOS). The safety system will be installed outside: as a first approximation, the range of the system can be predetermined by assuming the rail convoy and the Hi-Rail pickup are in line of sight (Figure 9).

(1) $$Pr=Pt.Gt.Gr{(\frac{c}{4\pi d\mathrm{Fc}})}^{2}\;$$

where *Pt*: Transmitter power;

*Pr*: Signal power at the receiver;

*Gt*: Transmitting antenna gain;

*Gr*: Receiver antenna gain;

*c*: Propagation speed in the air;

Fc: Carrier frequency.

Fixing *Pr* as the sensitivity of the receiver (*Sr*), d becomes the range (*R*) of the telecommunication system:

(2) $$R=\frac{c}{4\pi \mathrm{Fc}}.\sqrt{\frac{Pt.Gt.Gr}{Sr}}.$$

The antennas used [11] are specific for railways. We mention this choice now, during the theoretical study, because we need the antenna parameters to go ahead with range calculation. These are omnidirectional UHF antennas with a gain of 6 dB tuned to 915 MHz. For the system, with the XTend radio-frequency modules manufactured by the company Digi and the associated high gain antennas:

Fc = 915 MHz; *Gt* = *Gr* = 6 dB; *Sr* = −100 dBm; *Pt* = 1 Watt.

At a rate transmission of 125 kbps, the sensitivity is −100 dBm for XTends boards: therefore, the ideal theoretical range of the system is from (2) of 328.565 km.

Obviously, the propagation of the signal will not be in the line of sight and without any obstacles. Therefore, we must go a little further in the modeling.

### 4.2. Loss Path Model with Ground Reflection

**Figure 10:**
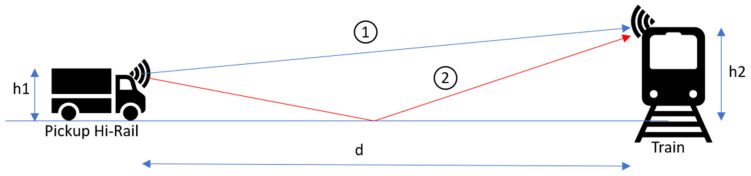
Ground reflection Train-Hi-Rail pickup.

Therefore, we consider a first-order reflection on the ground (Figure 10) with two trajectories:

- Direct line;
- With a reflection on the ground.

This “ground reflection” model is suitable because the transmitter and receiver antenna are not placed at the same heights. The Hi-Rail pickup antenna is at h1 = 2.5 m, while the locomotive antenna is h2 = 4.572 m or 15 feet.

The expression of the range becomes:

(3) $$d={\left[\frac{Pt.Gt.Gr.h2^{2}h1^{2}}{Sr}\right]}^{1/4}$$

The sensitivity of the receiver located on the Hi-Rail pickup is:

*Sr* = Eb.Rb = Eb/No. Rb.No = (Eb/N0).Rb. (k.Tsys) (4)

where Eb is the energy of a bit, Rb is the bit rate, Tsys is the noise temperature of the receiver, and k is the Boltzmann constant. For XTend modules, when the tests will be achieved, Rb will be set at 125 kbps with XCTU software, which afford to set the radio frequency modules’ parameters. Eb/No is the signal-to-noise ratio obtained from the bit error rate (BER) [12]. The modulation type for the XTend radio-frequency modules is non-coherent FSK type.

**Figure 11:**
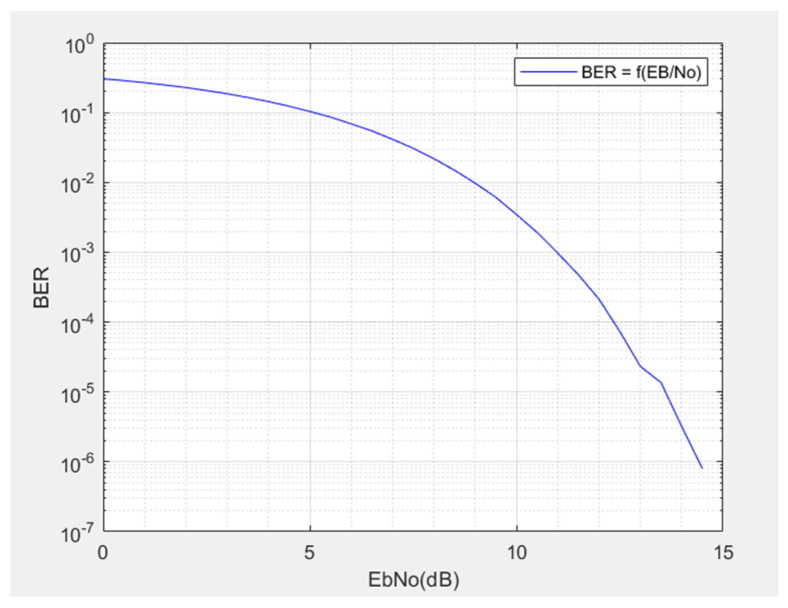
BER as a function of signal to noise ratio.

The MATLAB modeling [13] of the communication between the locomotive and the Hi-Rail pickup with a FSK modulator [14], a FSK demodulator, and an additive Gaussian noise gives the curve of the Bit Error Rate (BER) as a function of the signal-to-noise ratio (Figure 11). The telecommunication system is considered reliable for this modulation type if the bit error rate is set at 0.0001, so a signal-to-noise ratio of 12.5 dB. Therefore, Equation (4) gives a sensitivity of 1 × 10^−15^ Watt. Then, the range from Expression (3) is 37.93 km. This shows a strong reduction in the range of the rail telecommunication system compared to an ideal range obtained with the “Loss Path” model. However, it is necessary to further specify the system’s range, considering the multiple reflections of the transmitted signal.

### 4.3. Fading Model

#### 4.3.1. Fading Due to Time Spread

The reality of the propagation medium is a little more complex: the signal transmitted by the locomotive undergoes multiple effects as it travels to the Hi-Rail pickup. It undergoes reflections, diffraction, and scattering. This phenomenon is called multipath fading [15]. Reflection must be considered when the size of the objects on which the wave is reflected is greater than the signal’s wavelength, which is 33.33 cm for our carrier frequency at 915 MHz. Alert signs on the railway track have large dimensions compared to 33.33 cm: therefore, the reflections of the wave must be considered. When small objects of sizes less than the wavelength are present, the transmitted signal may be subject to scattering. When the signal encounters obstacles of small dimensions compared to its wavelength, a phenomenon is observed corresponding to the superposition of many random diffractions. Then, the incident wave is processed statistically. In our study, the rail convoy moves through a forest with “spruce”-type conifers. The dimension of thorns and other leaves present on deciduous trees is less than 33.33 cm: therefore, the phenomenon of scattering must be considered. Likewise, the edges of certain track signs will create a diffraction phenomenon on the transmitted wave.

The three phenomena (reflection, diffusion, diffraction) will create different signals arriving at the receiver with a time delay and different amplitudes. The resulting signal at the receiver is the sum of these signals. We can imagine in the worst case that this sum taking account of multipath delays would create a destructive result, thus condemning the reception of the signal transmitted by the locomotive. This multipath fading will degrade the transmission of information. There are two predominant statistical models to highlight this phenomenon:

- Rician model [16];
- Rayleigh model [17].

The Rician model involves multiple paths but also the main path called line of sight. In our study, there is no direct path between the locomotive and the Hi-Rail pickup due to the evolution of the railway in a wooden environment with small hills. Therefore, to model the phenomenon of multipath fading, the statistical model used is Rayleigh. As a reminder, the Rayleigh probability density is:

(5) $$fr=.e^{-r^{2}/2.\alpha^{2}}\;$$

where the effective value (RMS) of the distribution is $\sqrt{2\alpha }$. Then, Gaussian white noise representing the noise at the receiving antenna is added to obtain the complete model of the telecommunication channel.

In MATLAB [18], the Rayleigh communication channel between the locomotive and the Hi-Rail pickup is inspired by Willis [19]. The received signal is modeled as follows:

(6) $$x\left(k\right)=\overset{N}{\underset{i=1}{\sum }}path\left(k,i\right)\;\;\;$$

where

*N*: number of paths;

Contribution of the ith multipath signal:

path(k,i)=Ai.x(k−di) 

*Ai*: Gain of the ith path;

*di*: delay of the ith sample.

**Figure 12:**
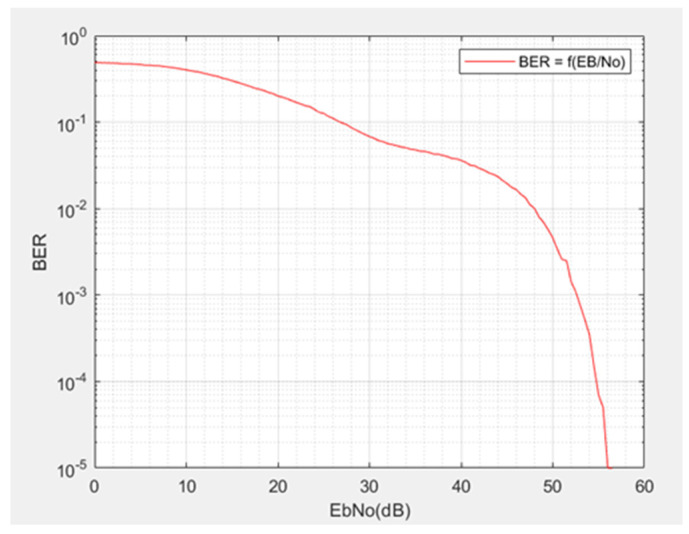
BER as a function of signal to noise ratio.

This Rayleigh model is a digital finite impulse response (FIR) filter. The additive noise is a Gaussian white noise (AWGN channel in MATLAB) [20,21], and the Rayleigh channel is the “SISO channel” block: this last block is configured for four paths for the signal transmitted with delays (τ) of respectively 0 μs, 8 μs, 12 μs, and 16 μs, which is usually used for outdoor telecommunication systems. These values correspond to distances traveled for the incident wave of 0 km, 2.4 km, 3.6 km, and 4.8 km, respectively. First, the FSK transmitter is set to 125 kbps. It is followed by the additive white noise channel and Rayleigh [22] channel. Then, the FSK demodulation block intervenes, and a comparison is made between the random binary sequence transmitted and the one received at the end of the telecommunication channel modelized. The MATLAB script produced allows us to obtain the curve of the BER as a function of the signal-to-noise ratio (EbNo) (Figure 12). The curve characterizes a degradation of reception. However, to obtain a successful reception with a BER of $10^{-4}$, the signal-to-noise ratio must be increased to 54 dB.

Thus, Expression (4) gives a sensitivity of −78.63 dBm, and finally, according to (3), the range becomes 3.5 km. Therefore, the multiple paths taken by the locomotive’s signal strongly affect the range of the railway telecommunication system. In general, when designing a wireless telecommunications system [23], a margin is defined for the sensitivity of the receiver, which is known as the fade margin. In the study of the railway telecommunication system, the sensitivity is −100 dBm, with a fading margin of 5 dB. Therefore, the sensitivity of the receiver is fixed at −95 dBm. For closed environments, the fading margin is between 6 and 10 dB.

To confirm this result, let us discuss about the spreading delay value $\left(\sigma_{\tau }\right)$ parameter representing the RMS of all multipath propagation time delay. The propagation channel has a bandwidth frequency parameter, which is coherence bandwidth and also known as Bc. Bc is often defined as 1(2.στ) : if the signal bandwidth is less than Bc, the propagation channel is flat in frequency. This means that $\sigma_{\tau }$ is much smaller than the symbol duration, and therefore, there will not be any intersymbol interferences (ISI). In the opposite direction, if the signal bandwidth is larger than the coherence bandwidth, it means that the symbol duration is lower than $\sigma_{\tau }$, and therefore, there will be ISI, which might degrade the transmission. With the values used earlier for τ in MATLAB simulations, $\sigma_{\tau }$ is equal to 5.916 μs. As the XTends module’s transmission rate is 125 kbps, the symbol duration is 8 µs, and the coherence bandwidth then equals 84.5 kHz: therefore, symbol duration and $\sigma_{\tau }$ are very closed, meaning that there will be ISI, and this confirms the performed MATLAB simulations results.

It is now interesting to consider the influence of the movement of the receiver, which will be the case with the Hi-Rail pickup leading to the Doppler effect.

#### 4.3.2. Doppler Effect

The Doppler effect is also a fading effect: when the receiver or an object inside the propagation channel is moving, it creates the Doppler effect. The consequence is a carrier frequency shift. The maximum frequency variation is the Doppler band Bd. Everyone has listened to the sound of an ambulance when it approaches and when it leaves: this is the Doppler effect. If the receiver is in motion, the propagation delay of the electromagnetic wave will therefore increase or decrease. The coherence time (Tc) of the propagation channel is the time over which the channel is not varying. This delay is equal to 1(2.Bd): if Tc is higher than the symbol duration (Ts), it means that the propagation channel is time invariant, and then, the Doppler effect is low. On the opposite, if Tc is lower than Ts, the propagation delay is variant over time, and therefore, the fading will be fast. The frequency shift ($f_{d}$) due to the Doppler effect is:

(7) $$\mathrm{fd}=\frac{v_{r}}{\lambda }\times \cos\left(\theta \right)=\mathrm{Fc}\times \frac{v_{r}}{v_{p}}\times \cos\left(\theta \right)$$

where *v* is the receiver velocity, λ is the wavelength of the electromagnetics wave, $v_{p}$ is the propagation velocity, θ is the angle of the electromagnetic wave when it arrives at the receiver, and Fc is the carrier frequency. Therefore, the maximum Doppler frequency shift is $\mathrm{Fc}\times \frac{v_{r}}{v_{p}}$, and the carrier frequency will be in the interval:

[Fc − vr/λ ; Fc + vr/λ ].(8)

The locomotive and the Hi-Rail pickup are most of the time in motion. It is legitimate to think that there will be a Doppler effect that might degrade the quality of the transmission. If we consider the speed (vr) of the Hi-Rail pickup relative to the train, it is as if the Hi-Rail pickup, therefore the receiver, was moving with a relative vr speed. In the study, if we consider a relative speed vr maximum of 5 km/h, the carrier frequency will vary then in the interval [915 MHz–4.2 Hz; 915 MHz + 4.2 Hz]. Therefore, the frequency variation ΔF is equal to 4.2 Hz or 0.00459 ppm. The XTend modules used in the receiver tolerate a frequency shift of 40 ppm: the Doppler effect will have no impact on this railway telecommunications system. The conclusion is the same if we calculate the coherence time Tc: Tc = 1/(2.Bd); then, 60 ms. For a data rate at 125 kbps, we have calculated earlier that the symbol duration value was 8 µs: therefore, the coherence time is much longer than the symbol duration. Even if we think of the case when the locomotive is moving and the Hi-Rail pickup is stopped: the relative speed is higher, and this is the worst case. vr equals 35 km/h (these are the rules of Transport Canada on this type of railroad), and then, the frequency variation interval is [915 MHz − 29.68 Hz; 915 MHz + 29.68 Hz] which gives a Doppler band to 59.36 Hz and then a coherence time of 8.42 ms, which is much higher than 8 µs (duration time). Even in this last case, the Doppler effect has no negative impact.

In conclusion, the Doppler effect due to the Hi-Rail pickup relative velocity does not affect the railway telecommunications system.

Now, let us consider the influence of the geographical and atmospheric criteria specific to the implementation area.

### 4.4. Irregular Terrain Model (ITM)

The ITM, better known as the Longley–Rice model [24], considers terrain (forest, city, mountain) and atmospheric data. It is a point-to-point statistical model that imposes antenna heights of at least 50 cm. It is suitable for ranges beyond the kilometer. The heights of the antennas of the Hi-Rail pickup and the locomotive are over 50 cm, and the distances separating the Hi-Rail pickup from the locomotive will always be over 1 km. Therefore, the model is very suitable for railway telecommunication system study.

The Radio Mobile telecommunication software uses this model to carry out coverage studies and link budgets. The images below show connection reports at various locations on the railway track between Emeril and Schefferville where the final system will be tested. A locomotive station has been created: it has an antenna height of 4.572 m, an omnidirectional antenna with a gain of 6 dB, a transmission power of 1 watt corresponding to the power of the XTend modules of the telecommunication system, and a 1 dB attenuation due to the connection losses and the length of the cable connecting the transmitter to the antenna. At the same time, a Hi-Rail pickup station has been implemented: it has an antenna height of 2.5 m, an omnidirectional antenna with a 6 dB gain, and a sensitivity of −100 dBm corresponding to that of the XTend modules used. As for the previous station, the losses generated by the connections and the cables (linking the antenna to the receiver) have been defined at 1 dB. The two stations have been teamed up to create a network.

**Figure 13:**
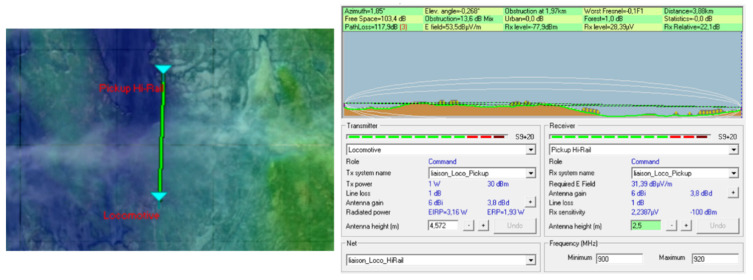
Budget link of the network located north of Emeril.

The simulation results in Figure 13 correspond to a network deployment on the railway north of Emeril. The image on the left shows the network with the locomotive as the 915 MHz transmitter and the Hi-Rail pickup as the receiver. The image on the right shows the locomotive and the Hi-Rail pickup with the topology of the area. It also shows the budget link: the distance between the two network elements is 3.88 km, and compared to the sensitivity of −100 dBm of the receiver, there is a margin of 22.1 dB. Therefore, communication is going perfectly.

**Figure 14:**
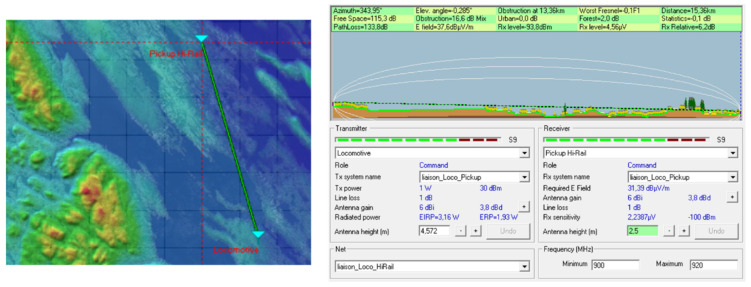
Budget link at Menihec on the railway.

Figure 14 shows another budget link for the locomotive–Hi-Rail pickup network near the place called “Menahec” on the railway track. The locomotive and the Hi-Rail pickup are 15.39 km apart, and communication is right, since the power margin at the receiver (Hi-Rail pickup) compared to its sensitivity is 6.2 dB.

**Figure 15:**
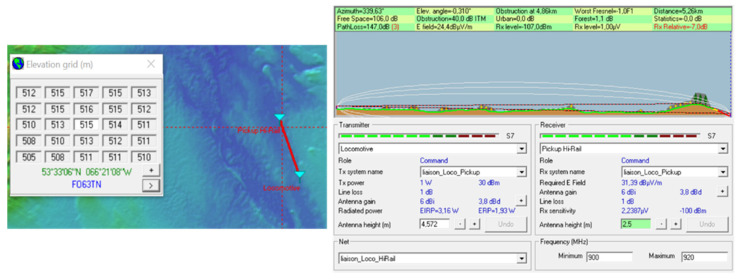
Negative budget link between Emeril (Labrador) and Schefferville (Quebec) on the railway.

Finally, Figure 15 shows a negative result: the network was placed halfway between Emeril and Schefferville. It appears from the picture on the right that a hill of about 514 m prevents the signal propagation transmitted by the locomotive: the power balance is negative since the receiver (Hi-Rail pickup) receives a power lower by 7 dB than its sensitivity.

## 5. System Tests on Site

### 5.1. Design Decisions

**Figure 16:**
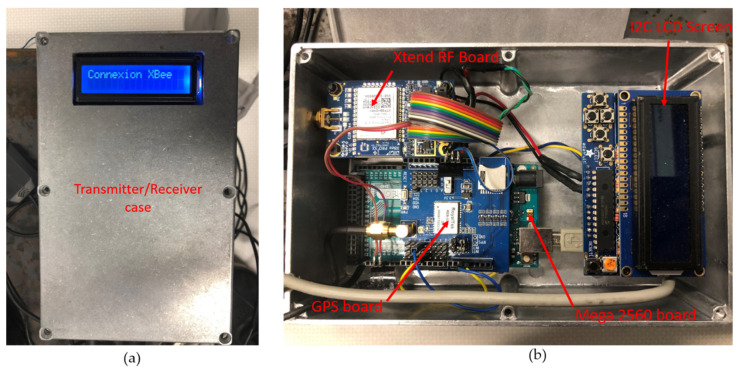
In (**a**) the Receiver/Transceiver case; (**b**) the inside of the Receiver/Transceiver.

The transmitters and receivers (Figure 16) have been designed around an Arduino Mega 2560 board with a GPS module and the XTend radio frequency module. The GPS board and the XTend board dialog with the 2560 microcontroller by two physical serial ports (RS232). An LCD screen connected in I2C was also installed in each of the cases (transmitter and receiver) to display results similar to the distance between the train and Hi-Rail pickup.

**Figure 17:**
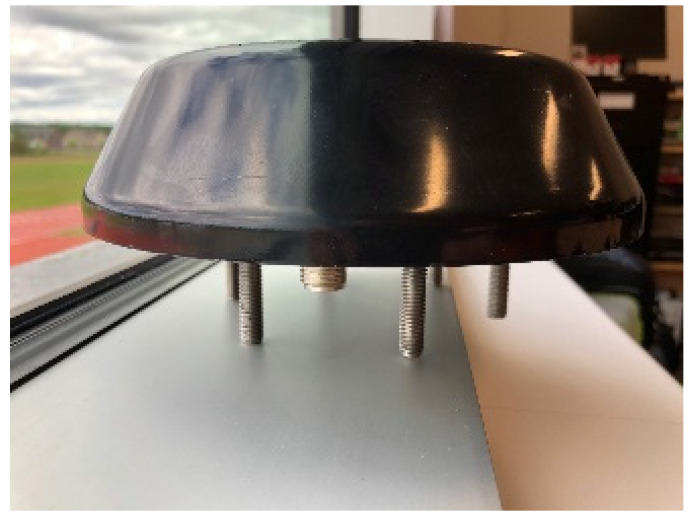
Specific UHF railway antenna.

**Figure 18:**
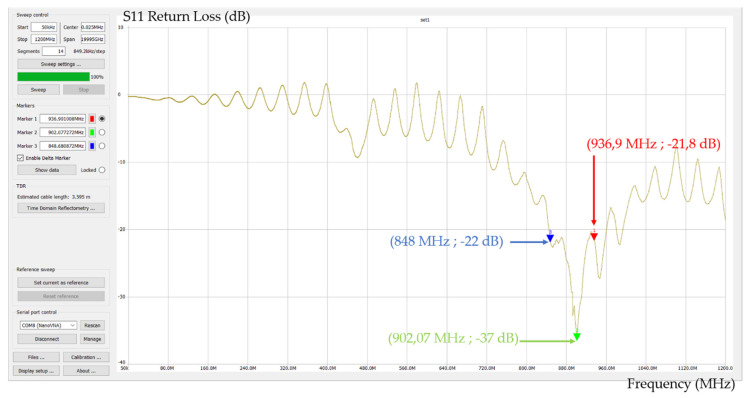
Antenna S11 return loss measured with network analyzer.

To confirm that the specific antennas (Figure 17) chosen are tuned at the right frequency, we have used a network analyzer to analyze the antenna S11 return loss (Figure 18): the result is quite coherent because the antenna center frequency is 902.07 MHz, and the bandwidth is large enough to work at 915 MHz.

**Figure 19:**
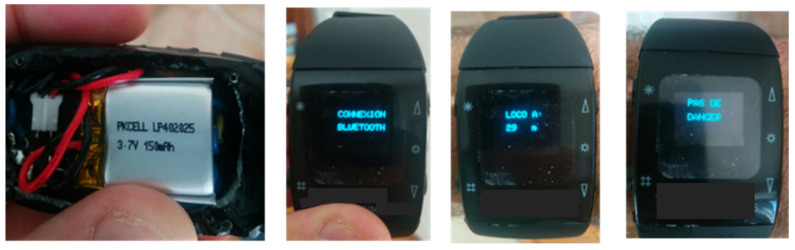
Watch connected in Bluetooth to the Hi-Rail pickup.

The watch has been fully developed (Figure 19): this watch should be resistant in hard-working conditions and it must be open source if later, other functionalities (hardware and software) are helpful to implement. Therefore, it is not possible to use ready-made watches. The heart of the watch is a microcontroller 32u4 driving a 0.66” TFT screen by SPI connection. There is also a buzzer and a very small Bluetooth board to receive the distance from the receiver located in the Hi-Rail pickup. The Bluetooth Low Energy (BLE) is the solution used in the connected watch: the battery life is important, and BLE gives this possibility to extend it. The battery is a lithium-ion 3.7 volts and 150 mAh: note that a technology such as Wifi is never used for the connected watch because of the high consumption of this technology. As the employees are working around the Hi-Rail pickup, the Bluetooth range is enough.

The following sequences were programmed and are displayed:

- Bluetooth connection: when the watch is switched on, it looks for pairing with the receiver in the Hi-Rail pickup;
- No danger: meaning that the locomotive is outside a distance of 10 km;
- The distance and a slowly tonality when the locomotive is located at a distance between 7 and 10 km;
- The distance and a medium tonality when the locomotive is located at a distance between 3 and 7 km;
- The distance and a very high-pitched sound when the locomotive is inside 3 km and also a flashing display.

### 5.2. Railway Telecommunication System Test Procedure

**Figure 20:**
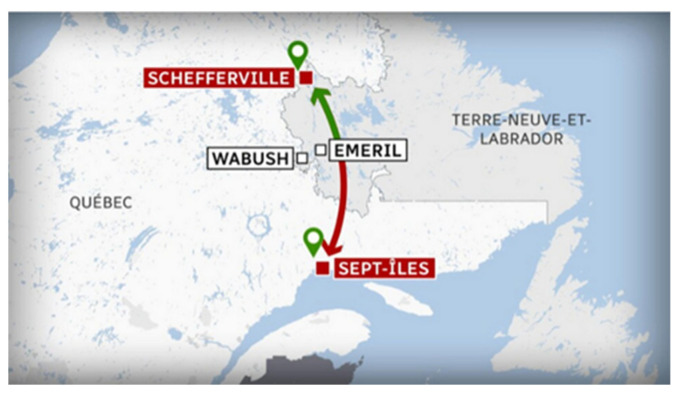
Illustration of Tshieutin company railway in northern Quebec.

The tests took place in northern Quebec between Emeril and Schefferville on the Innu Tshiuetin company railway (Sept-Îles, QC, Canada) (Figure 20) in October: the weather was very nice during the test session. This company is an autochtone company with low resources, and so it was necessary to design an open-source system.

**Figure 21:**
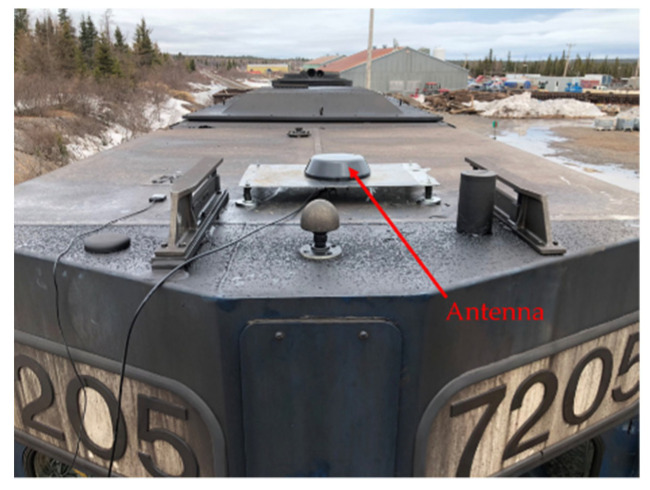
Antenna attached to the locomotive roof.

**Figure 22:**
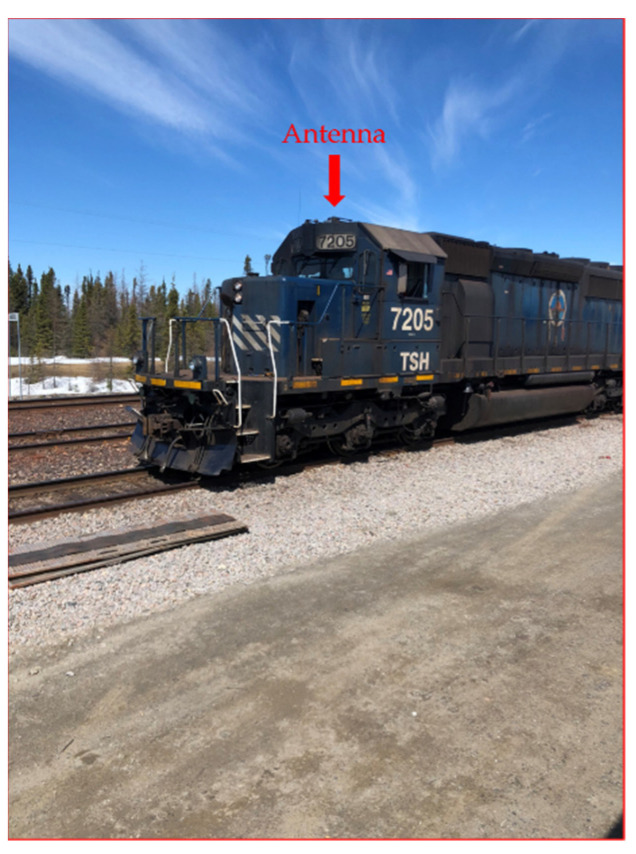
Antenna fixed to the top of the train.

**Figure 23:**
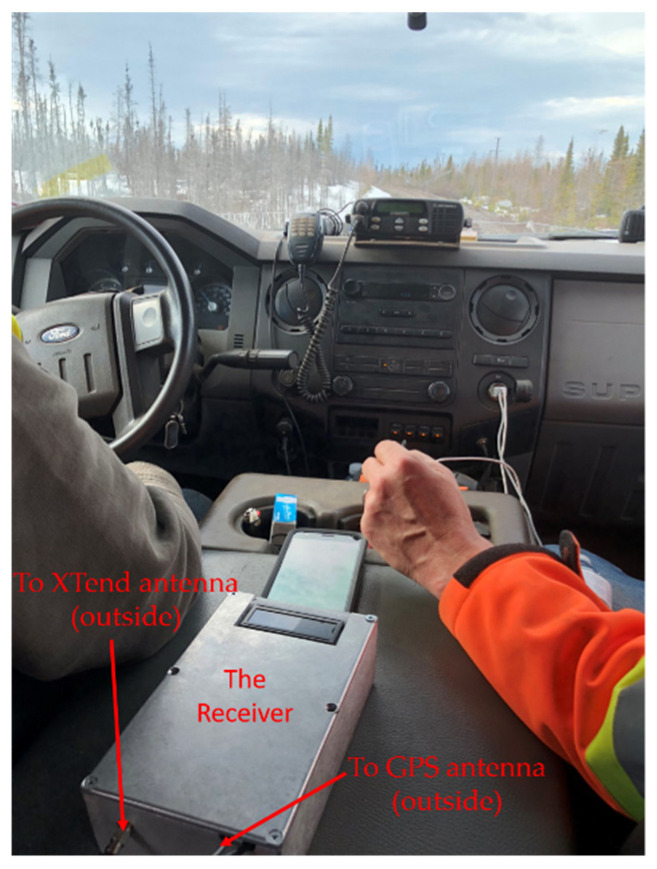
Receiver in the Hi-Rail pickup.

The antennas were mounted on brackets with magnets to easily fix them on the top of the locomotive (Figure 21 and Figure 22) and the top of the Hi-Rail pickup in Schefferville station. The transmitter was installed in the cockpit of the locomotive and the receiver was installed in the Hi-Rail pickup (Figure 23).

The 250 km of rail tracks linking Schefferville to Emeril with the receiver in the Hi-Rail pickup and the transmitter in the locomotive were completed in 7 h with a permanently modulated Hi-Rail-pickup to locomotive distance, and the tests resumed the next day on the way back between Emeril and Schefferville. It should be noted that later, during the deployment phase, the XBee technology will be of interest: each locomotive and each Hi-Rail pickup will have the address of their XBee module, and thus, we can create an expandable Mesh network. This will be especially useful with a growing fleet of Hi-Rail pickups.

### 5.3. Tests Results

The results of the tests are summarized in the table (Table 2) below:

The average range obtained is around 3 km, and ranges of 10 km have been achieved. As expected, the lower ranges were obtained in places where there were small mountains. The results obtained are in coherence with the large numbers of simulation achieved with Radio Mobile. The Tshiuetin company wanted an alert system with a range of 2 km, and as such, the tested solution is right. Likewise, the Bluetooth-connected watch was also tested with a Hi-Rail pickup stopped on the railway while the locomotive was approaching. These tests were conclusive, and the range obtained between the receiver (pickup Hi-Rail) and the watch is not surprising: it is 20 m with the BLE boards installed in the watch and in the receiver. Note that there will not be any interferences due to the presence of a locomotive and two Hi-Rail pickups, for example. We have achieved some interference pollution tests with an XBee transmitter and two XBee receivers located in a very close environment: the transmitter was sending data to both XBee receivers: the error measured was 0.01%, which means that the XBee modules do not pollute each other: the result is not surprising when you consider that Space X’s rocket uses XBee modules for internal communication in an environment with lots of interference.

## 6. Conclusions

The aim of our research project was to design and to validate a railway safety system for an isolated environment and especially for Nordic trains in Quebec based on IEEE 802.15.4 protocol. This new low-power rail security system has been validated on the rail tracks of northern Quebec, and the results obtained define a mean range of 3 km and a maximum range of 10 km. The railway company Tshiuetin has approved the results of the tests. It is certain that a greater range between the locomotive and the Hi-Rail pickup could be obtained with the same power by placing the antennas at higher heights. However, the locomotive crosses tunnels, which makes this eventuality impossible. It is also necessary to imagine that the winter conditions are very hard with snow accumulation and ice on the locomotives. Therefore, it is required to have a compact and aerodynamic antenna: it will be necessary to test the system again during the winter and the very hard climate conditions of the northern Quebec. The methodology we used for our tests could have been improved if we had had full control over the movement of the locomotive, as it has been the case with the Hi-Rail pickup we used. Nevertheless, the company could not monopolize a convoy for us during our tests, as the mines continue to produce iron ore that fills the rail cars to be driven to the port without any delay. Therefore, we had to adapt our methodology to the situation: despite everything, this choice of methodology allowed us to test deeply our telecommunication system.

The next step requested by the Tshiuetin company confirms the success of this first project: the company wants to make the system bidirectional so that the driver of the locomotive is warned of the presence of employees on the railway in this isolated environment where there is no internet connection and no electricity network. Another avenue for the development of an ultra-modern solution in this isolated environment would consist of mapping the GPS coordinates of the Hi-Rail pickup transmitted by the XBee system on an offline tablet located in the locomotive. Thus, the driver of the locomotive could see in real time the position of the employees on the track.

After developing this geolocation system with an offline tablet, another paper will be written and submitted to Sensors. Finally, the prospects for improving the safety for the company based on this project are significant, especially in this isolated environment. Our system gives a very important first layer for a railway safety system with the possibility of implementing lots of other functionalities. Every difficult step of designing the system has been achieved with a scientifical process: this gives lots of complete and precious details to take into account for any scientists who want to develop a telecommunication system in those specific places that are isolated environments on the earth.

Note also that some important railways companies that drive iron from north Quebec follow with lots of interest the evolution of our railway security system to implement in the near future on their tracks.

## Figures and Tables

**Table 1:** Comparison of the different wireless technologies.

Technology	Frequency (MHz)	Range	Rate	Operating Conditions	Suitable for Isolated Environment with High Range
LoRa	915	25 km	[290 bps; 50 kps]	Internet Connection Needed	No
XBee UHF	915	64 km	50–250 kbps	No Condition	YES
XBee	2400	100 m	50–250 kbps	No Condition	No
LTE	700; 850 1700/2100 2600	<30 km per cell	<75 Mbps (uplink) <300 Mbps	LTE Base Stations Needed	No
Bluetooth	2400	20 m	54 Mbps (BLE 5)	No Condition	No
Z-Wave	915	400 m	100 kbps	No Condition	No
Wifi 1	2400	90 m	[450 Mbps; 600 Mbps]	No Condition	No
Wifi 2	5000	30 m	<1.35 Gbps	No Condition	No

**Table 2:** Summary of results planned (before tests) and obtained (after tests).

Results	Modelizations and Simulations	On Site (Railway Track)	Wishes of the Railway Company
Mean Range	3.5 km	3 km	2 km
Maximum Range	15.36 km	10 km	3 km

## Data Availability

Generated during the study.
